# Microbial nutrient niches in the gut

**DOI:** 10.1111/1462-2920.13659

**Published:** 2017-02-03

**Authors:** Fátima C. Pereira, David Berry

**Affiliations:** ^1^ Department of Microbiology and Ecosystem Science, Division of Microbial EcologyUniversity of ViennaAlthanstrasse 14Vienna1090Austria

## Abstract

The composition and function of the mammalian gut microbiota has been the subject of much research in recent years, but the principles underlying the assembly and structure of this complex community remain incompletely understood. Processes that shape the gut microbiota are thought to be mostly niche‐driven, with environmental factors such as the composition of available nutrients largely determining whether or not an organism can establish. The concept that the nutrient landscape dictates which organisms can successfully colonize and persist in the gut was first proposed in Rolf Freter's nutrient niche theory. In a situation where nutrients are perfectly mixed and there is balanced microbial growth, Freter postulated that an organism can only survive if it is able to utilize one or a few limiting nutrients more efficiently than its competitors. Recent experimental work indicates, however, that nutrients in the gut vary in space and time. We propose that in such a scenario, Freter's nutrient niche theory must be expanded to account for the co‐existence of microorganisms utilizing the same nutrients but in distinct sites or at different times, and that metabolic flexibility and mixed‐substrate utilization are common strategies for survival in the face of ever‐present nutrient fluctuations.

## Introduction

The gut microbiota is the community of commensal, beneficial, and pathogenic microorganisms that inhabit the gastrointestinal tract of humans and other animals. Forces that shape the composition of the gut microbiota, as well as other microbial communities, can include stochastic processes such as dispersal, genetic diversification, and ecological drift. However, deterministic interactions between species, individuals, and the environment also create defined niches and thereby influence community composition. The ecological niche was described by Charles Elton in 1927 as the ecological component of a habitat related to an organism's tolerances and requirements, with a focus on its nutrient‐foraging capacities (Elton, 1927). Elton's niche concept was later generalized by Hutchinson, who envisioned a niche as a multidimensional space of resources and environmental conditions that together define where an organism can survive and grow (Hutchinson, 1957). Importantly, the range of possible conditions under which a species can grow – referred to as its fundamental niche – can be much broader than its actual niche. In this realized niche, the overall potential of a species to exploit its fundamental niche is limited by factors such as environmental conditions, nutrient availability, and the presence of competitors, predators, or phages. Species with overlapping fundamental niches can co‐exist by adjusting to each other and segregating their realized niches in a process called niche differentiation. If niche differentiation is not possible, the competing species most well‐adapted to the niche would be expected to outcompete and completely exclude the inferior competitor.

The gut environment and potential niche space can be determined by the host in a variety of ways. The host immune system can act as an environmental filter to limit or expand available niches. However, niche space in the gut is thought to be largely determined by the abundance and types of nutrients derived from host diet as well as secreted into the gut by the host. This review will focus on key aspects of nutrient niches in the gut microbiota, considering the gut as a dynamic ecosystem in which spatial and temporal heterogeneity in the nutrient landscape shape the composition of the microbiota.

## Survival of the fittest

Hundreds to thousands of co‐existing species of microorganisms inhabit the mammalian gut (O‘Hara and Shanahan, 2006). The mechanisms underlying the assembly and structure of the microbiota are, however, far from being fully understood. Due to its simplicity, the neutral theory of community assembly has been proposed as a reasonable starting point, or ‘null model’, to explain microbiota assembly. Neutral theory assumes that all species are equally‐fit competitors and that the presence and abundance of a species in an ecosystem is shaped only by stochastic processes such as dispersal (i.e. movement of organisms across space) and ecological drift (i.e. random fluctuations in population size) (Caswell, 1976; Hubbell, 2001; Rosindell *et al*., 2011). Under these conditions, organisms in the community are randomly lost and are replaced at random by individuals from within the community or by immigration of individuals from outside the community. The observation that temporal fluctuation of taxa is not accompanied by major differences in function (as determined with metagenomics) (Thaiss *et al*., 2014) could be taken as evidence of stochastic fluctuations of equally‐fit species. High levels of dispersal leads to accumulation of diversity into local microbial communities, thus increasing alpha diversity (Chase and Myers, 2011), as well as to the homogenization of communities, thus decreasing beta diversity (Cadotte, 2006). Increased alpha diversity and decreased beta diversity are observed in the gut microbiota of tribal populations in comparison to non‐tribal populations, suggesting that better sanitation and other hygienic practices associated with industrialization might affect the dispersal of gut organisms and thereby influence the composition of the gut microbiota (De Filippo *et al*., 2010; Yatsunenko *et al*., 2012; Schnorr *et al*., 2014; Martinez *et al*., 2015). Neutral theory has been used with some success to explain the assembly of microbial communities in diverse environments, including host‐associated microbiomes (Sloan *et al*., 2006; Woodcock *et al*., 2007; Costello *et al*., 2012; Jeraldo *et al*., 2012; Venkataraman *et al*., 2015; Burns *et al*., 2016; Sala *et al*., 2016). For example, the composition of the healthy lung microbiota can be explained by dispersal of bacteria from other body parts (Venkataraman *et al*., 2015). However, models based on neutral theory could only incompletely explain the composition of the gut microbiota for several domestic vertebrates, with deviations particularly apparent for the most abundant species (Jeraldo *et al*., 2012; Sala *et al*., 2016). In a screening of stool samples from hundreds of individuals residing in the United States, only one sample had a microbiota composition consistent with neutral theory, strongly suggesting that, despite potential dispersal limitations, deterministic processes are key in shaping the microbiota (Li and Ma, 2016). A clear illustration of the importance of deterministic processes such as niche adaptation is that although germ‐free mice can be stably colonized with microbial communities collected from diverse habitats (soil, microbial mats, termite gut, fish gut, and human skin, tongue and gut), these communities are outcompeted and driven to extinction when challenged by an invading mouse gut microbiota (Seedorf *et al*., 2014). Also of note, for many of the tested allochthonous communities the gut environment selected for organisms having polysaccharide utilization genes. Specifically, the ability to degrade starch, a major component of the laboratory mouse diet, largely determined colonization success, highlighting the importance of the nutrient landscape as a driving force in microbiota assembly.

**Figure 1:**
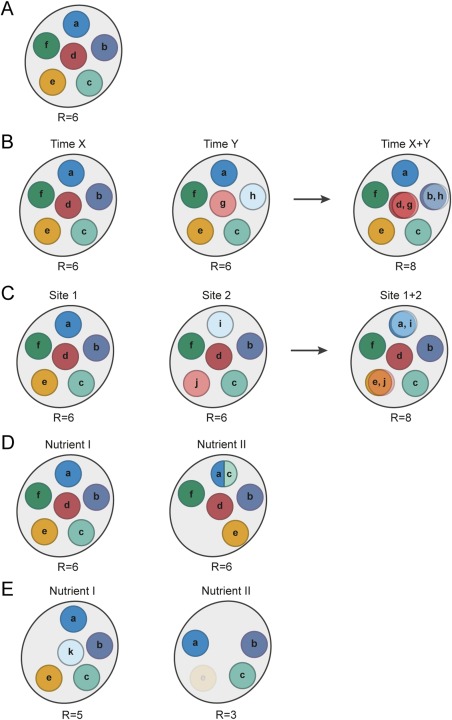
Niche‐space diagrams representing nutrient niche concepts related to the abiotic (A‐C) and biotic (D, E) environment. The total niche space is shown as a large ellipse and the realized niche for each species is represented as a circle. Species are represented by letters *a*‐*k*. A. Freter's concept of nutrient niches considering well‐mixed nutrients and equilibrium conditions: each species occupies a preferred nutrient niche. B. Exploitation of the same nutrient niche by different species under non‐equilibrium conditions (i.e. unbalanced growth): at different times *g* and *h* have the same nutrient niche as *d* and *b* respectively. C. Extension of Freter´s theory assuming spatial structuring (i.e. Restaurant hypothesis): the same nutrient niche can be used by different species (e.g. *a* and *i*, *e* and *j*) at distinct sites. D. Niche switching and niche partitioning due to metabolic flexibility of species. Changes in the nutrient landscape force *e* and *c* to switch their niches, and *a* and *c* partition the previous niche of *a*. E. The effect of obligate and facultative dependencies, as well as keystone species. Nutrient fluctuations lead to disappearance of a keystone species *k*. Species *e* is completely dependent on the activity of *k* and goes extinct, while *a* is able to switch its niche and persist in the absence of *k*. R = species richness.

**Figure 2:**
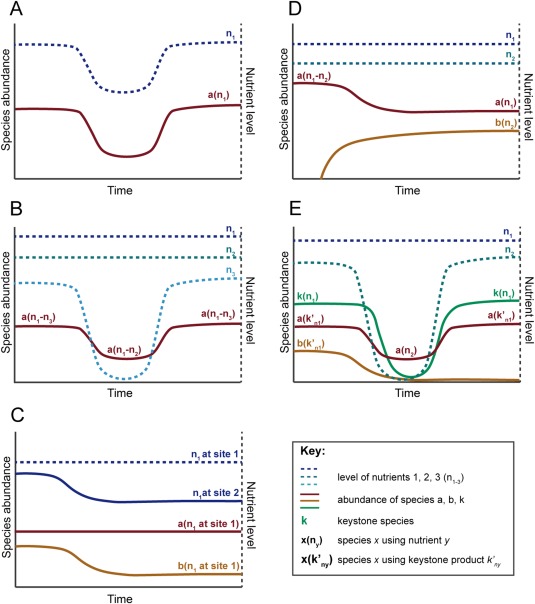
Consequence of nutrient fluctuations on species abundance considering different models of nutrient niches. A. In Freter's nutrient niche model species *a* abundance is determined by a single limiting nutrient (n_1_). B. In the mixed substrate utilization model *a* uses several nutrients (n_1‐_n_3_) simultaneously and changes in the level of single nutrients has minimal effects on its abundance. C. If there is a heterogeneous distribution of nutrients (i.e. Restaurant hypothesis) *a* and *b* use the same nutrient (n_1_) at different sites and are affected by local nutrient levels. D. Niche partitioning: Species *b* invades and outcompetes *a* for nutrient n_2_. E. Obligate and facultative dependencies with keystones: *b* is completely dependent on the nutrient *k’_n1_* provided by keystone species *k* and goes extinct when *k* is absent, while *a* is metabolically flexible and can switch to another nutrient (n_1_) when *k* is absent.

Though various factors such as host genotype, immune status, and health state can affect the composition of the gut microbiota, the primary driver appears to be the composition and intake levels of host diet (Turnbaugh *et al*., 2009; Wu *et al*., 2011; David *et al*., 2014b; Zarrinpar *et al*., 2014; Carmody *et al*., 2015). In 1983, Rolf Freter formulated the nutrient niche theory, which asserts that ecological niches in the gut are defined by available nutrients and that a species can only colonize if it is able to most efficiently use a particular limiting nutrient (Fig. 1A) (Freter *et al*., 1983a, 1983b). The levels of one, or maximally a few, limiting nutrients would therefore be predicted to dictate the abundance of each species (Fig. 2A). Nutrient niche theory is supported by observations in gnotobiotic mouse models that the concentration of individual dietary components can explain the relative abundance of each member of a 10 species community (Faith *et al*., 2011) and that *Bacteroides cellulosilyticus* levels are controlled by levels of dietary arabinoxylan (Wu *et al*., 2015). The nutrient niche theory is also supported by numerous *in vivo* and *in vitro* diet supplementation studies showing that different prebiotics can target very specific organisms or groups of organisms in a complex gut community (Macfarlane *et al*., 2008; Ramirez‐Farias *et al*., 2009; Martínez *et al*., 2010; Walker *et al*., 2011; Ivarsson *et al*., 2014; Chung *et al*., 2016; Duncan *et al*., 2016). In a natural, fully‐developed gut microbiota, one might expect all available nutrient niches to be occupied. A new incoming species, whether it be a commensal or pathogen, should be unable to establish unless it can outcompete a resident species or a vacant niche arises due to a new component in the diet or elimination of a competitor, as may occur during antibiotic administration or inflammation. Supporting this notion, commensal *Escherichia coli* strain Nissle 1917 can outcompete *Salmonella enterica* serovar Typhimurium (*S*. Typhimurium) due to its superior iron uptake capacities (Deriu *et al*., 2013). Furthermore, it has been recently shown that an introduced strain of *Bifidobacterium longum* can colonize and persist in the human gut unless functionally‐similar organisms, which are presumably better competitors for certain carbohydrates, are already present (Maldonado‐Gómez *et al*., 2016). In some cases, more than one strain is needed to saturate potential nutrient niches available to incoming strains and thereby block colonization (Lawley *et al*., 2012; Stecher *et al*., 2013; Brugiroux *et al*., 2016). For instance, two commensal *E. coli* strains, HS and Nissle 1917, are required to prevent colonization of streptomycin‐treated mice by the pathogen *E. coli* EDL933 (Maltby *et al*., 2013). HS and Nissle 1917 utilize different subsets of sugars that can be used by EDL933. Though HS has the genetic potential to use ten different mucosal‐derived sugars, it actually only utilizes six of these *in vivo* (Maltby *et al*., 2013). Thus, the realized niche of HS differs from its fundamental niche. This example highlights that care must be taken in interpreting a species’ nutrient niche based on genome analysis alone, as this provides information about the fundamental, but not the realized, niche of an organism. Complementing genome analysis with transcriptomics proved to be crucial to understand how two strains of lactobacilli, *Lactobacillus reuteri* strain 100–23 and *Lactobacillus johnsonii* strain 100–33, both of which are able to use glucose and maltose, the two main fermentable carbohydrates in the mouse stomach, could cohabit in forestomach biofilms (Tannock *et al*., 2012). Despite having overlapping fundamental niches, both strains can co‐exist by restricting their realized niches and partitioning these resources, with 100–23 utilizing maltose and 100–33 utilizing glucose (Figs 1D and 2D). In a recent study of gene expression of pairs of co‐occurring human gut microbes, it was observed that for 41% of all pairs of species, the presence of one of the organisms was associated with an altered transcriptional profile in the other (Plichta *et al*., 2016). Transcriptional changes were most pronounced in genes involved in nutrient uptake and anaerobic respiration, suggesting that nutrient niche partitioning is a prevalent phenomenon in the human gut microbiota (Plichta *et al*., 2016).

## The dynamic gut

The restriction of an organism's realized niche in a complex community lends support to Freter's theory that the level of one or few nutrients controls the population size of any individual species. However, if a single limiting nutrient determines the success of a species it is surprising that day‐to‐day variations in diet do not destabilize the gut microbiota (David *et al*., 2014a). If nutrient niche theory is correct, successful species must have evolved to be versatile enough to switch their realized nutrient niche regularly. Alternatively, if a species was simultaneously utilizing multiple substrates (Kovarova‐Kovar and Egli, 1998), the loss of an individual substrate might have a minor effect on its abundance. *Salmonella*, for example, utilizes diverse nutrients *in vivo* (Steeb *et al*., 2013). While there may be substrates that are indeed irreplaceable, such as hydrogen for *Salmonella*, many organic carbon substrates are perhaps interchangeable and used in parallel, which would account for the stability of populations (Fig. 2B). For example, in a gnotobiotic mouse model, *B. cellulosilyticus* fitness was determined by 550 loci in its genome when mice were fed a high‐fat, high‐sugar diet, but only 34 loci were critical fitness determinants when mice were fed a low‐fat, high‐plant polysaccharide diet (Wu *et al*., 2015). This suggests that in a complex nutrient environment, such as a high‐plant polysaccharide diet, there is more possibility for mixed‐substrate utilization or niche switching. Taken together, these results indicate that while the population levels of some species may be controlled by a single substrate, others are likely involved in mixed‐substrate utilization or are versatile enough to switch nutrient sources depending on availability (Figs 1D and 2B).

Nutrient niche theory is based on the premise of balanced microbial growth, such as occurs in a steady‐state chemostat system. In most cases, however, dietary intake is not continuous and can vary widely in frequency, thereby creating temporal changes in the nutrient landscape. The gut microbiota of fasting or hibernating animals exhibits large fluctuations (Crawford *et al*., 2009; Sonoyama *et al*., 2009; Costello *et al*., 2010; Sommer *et al*., 2016). In Burmese pythons, fasting is associated with a loss in diversity and an increase in the abundance of *Bacteroides* and *Akkermansia*, most likely due to their ability to switch their metabolism from dietary glycans towards degradation of host‐derived compounds such as mucin (Costello *et al*., 2010). Similarly, an increase in *Akkermansia* was also observed in fasted hamsters (Sonoyama *et al*., 2009). In brown bears, hibernation leads to an increase in *Bacteroidetes*, though not of *Verrucomicrobia* (*Akkermansia*), and a decrease in *Firmicutes* and *Actinobacteria*, who presumably rely on the presence of dietary fibres (Sommer *et al*., 2016). In fasting animals, nutrient‐poor conditions may force many members of the gut microbiota to survive at low abundance or become dormant until the next meal. The slow intestinal transit induced by fasting or hibernation allows the persistence of dormant organisms inside the host, which can resume growth whenever nutrients conditions are again favourable. While fasting is a rather extreme case for most animals, even daily variation in dietary intake can affect the gut microbiota. For example, the intake of fibre‐rich foods in humans correlates positively with next‐day abundances of *Bifidobacteria*, *Roseburia spp*., and *Eubacterium rectale* (David *et al*., 2014a). Additionally, examination of gut microbiota in conventionally‐raised mice showed differential diel variation in microbial structure and function, with the majority of oscillating operational taxonomic units (OTUs) belonging to the family *Lachnospiraceae* (Leone *et al*., 2015). Temporal variations in the nutrient landscape, whether they be stochastic due to daily variation in diet or rhythmic such as meal frequency, create the conditions for non‐balanced microbial growth. What are the implications of non‐balanced growth in the gut? According to the r/K selection theory introduced by the ecologists Robert MacArthur and E.O. Wilson, under non‐equilibrium conditions competitive exclusion may not be reached and organisms able to grow more quickly but less efficiently on a preferred nutrient (i.e. r‐strategists) and organisms that grow more slowly but with higher affinity for a preferred nutrient (i.e. K‐strategists) can co‐exist with a balance in abundances that depends on the frequency of the nutrient fluctuations (Pianka, 1970). This would support a higher diversity and also allow organisms utilizing the same limiting nutrient to co‐exist (Fig. 1B). It may be that feeding frequency and gut transit time are key factors in determining the outcome of r/K selection. Indeed, longer gut transit times and increased stool consistency (which is positively correlated with transit time) are associated with higher microbial richness (Roager *et al*., 2016; Vandeputte *et al*., 2016), suggesting that K‐strategists are supported by longer periods of low nutrient conditions.

## Compartmentalized gut

**Figure 3:**
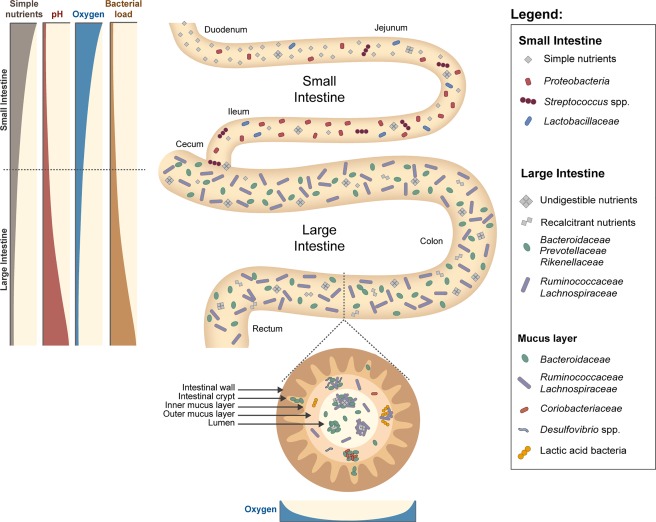
Spatial heterogeneity of the gut microbiota in the gastrointestinal tract. Gradients of pH and oxygen along the longitudinal axis limit the bacterial load in the proximal regions of the small intestine, whereas the large intestine carries high bacterial loads. Simple nutrients abound in the small intestine and sustain the growth of taxa able to effectively scavenge these compounds. In contrast, the large intestine is populated by taxa that can break down recalcitrant compounds. There is also spatial heterogeneity along the cross‐sectional axis of the intestine, with the mucus layer and the lumen harboring distinct microbial communities that reflect differences on nutrient availability. Fine‐scale spatial structuring is observed in both the mucus and lumen, with a heterogeneous distribution of nutrients sustaining different bacterial communities at particular sites.

Stool is often used as a proxy for the intestinal microbiota due to relative ease of sampling. However, the mammalian intestinal tract has multiple compartments with different physicochemical and nutrient conditions and, as a consequence, different microbial communities (Fig. 3). In the small intestine, rapid intestinal transit as well as higher levels of oxygen (He *et al*., 1999) select for fast‐growing facultative anaerobes that can compete with the host and other microorganisms for simple sugars. In the human ileum these include *Proteobacteria* (mainly *E. coli*) and *Streptococcus* spp. (Zoetendal *et al*., 2012; Donaldson *et al*., 2016) as well as *Bacteroidetes* and members of *Clostridium* clusters IX (*Veillonella* spp.) and XIVa, which may grow on fermentation products such as acetate and lactate that are secreted by abundant facultative anaerobes (Zoetendal *et al*., 2012). The murine small intestine is enriched in *Lactobacillaceae*, *Bacteroidales* and *Desulfovibrionaceae* (Gu *et al*., 2013; Donaldson *et al*., 2016). Simple, easily‐metabolizable nutrients are largely depleted in the small intestine by host absorption or microbial utilization and the vast majority of species that populate the large intestine are strict anaerobes that ferment complex polysaccharides and other refractory dietary compounds as well as secreted host compounds such as mucin. Lower levels of bile acids and secreted antimicrobial compounds as well as a less acidic pH in the large intestine also likely contribute to a higher cell density and diversity compared with the small intestine (Booijink *et al*., 2010; Zoetendal *et al*., 2012). Because of the higher density of cells in the large intestine, stool samples are generally considered to be representative of the colonic microbiota (Gu *et al*., 2013; Yasuda *et al*., 2015). The majority of organisms found in stool samples from healthy humans are facultative or obligate anaerobes belonging to *Firmicutes* and *Bacteroidetes* phyla (Donaldson *et al*., 2016). Similarly, the murine colonic microbiota includes members of the *Firmicutes* and *Bacteroidetes* such as *Ruminococcaceae*, *Lachnospiraceae*, *Bacteroidaceae*, *Prevotellaceae* and *Rikenellaceae* families (Nava *et al*., 2011; Gu *et al*., 2013). The capacity to degrade dietary fibres is a common trait shared by these taxa (Flint *et al*., 2012). However, the fermentation potential of different fibre types can be species‐ and strain‐dependent. For example, amendment of human stool with amylase‐treated wheat bran results in an increase in members of the *Lachnospiraceae* such as *Eubacterium xylanophilum* and *Butyrivibrio* spp. (Duncan *et al*., 2016). Dietary supplementation with resistant starch (RS) boosts the relative abundance of *Ruminococcus bromii* and *Eubacterium rectale* (for RS types 2 and 3) as well as *Bifidobacterium adolescentis* and *Parabacteroides distasonis* (for RS type 4) (Martínez *et al*., 2010; Walker *et al*., 2011). Inulin also increases the relative abundance of *B. adolescentis* as well as *Faecalibacterium prausnitzii* in humans (Ramirez‐Farias *et al*., 2009), while in rats the main utilizers of administered ^13^C‐labeled inulin are *Bacteroides uniformis*, *Blautia glucerasea*, *Clostridium indolis* and *Bifidobacterium animalis* (Tannock *et al*., 2014). These results highlight that dietary fibres can distinctively modulate the composition of the colonic microbiota.

Different communities are observed not only along the length of the intestinal tract but also along its cross‐sectional axis (Fig. 3). The epithelial tissue delimiting the lumen secretes a layer of mucus that is a nutrient source for some gut bacteria and supports an immature biofilm characterized by low cell density, with an estimated 10^5^−10^6^ cells in the mucus compared to 10^11^−10^12^ in the lumen (De Weirdt and Van de Wiele, 2015). Studies of human biopsies have reported that the colonic mucus layer is slightly enriched in some taxa such as *Lachnospiraceae*, *Ruminococcaceae*, *Bacteroidaceae* and *Coriobacteriaceae* (Ouwerkerk *et al*., 2013; De Weirdt and Van de Wiele, 2015; Lavelle *et al*., 2015). *Bacteroides* spp. and lactic acid bacteria including *Leuconostoc* spp., *Weissella* spp. and *Lactococcus* spp. were also found to be abundant in the mucosa‐associated microbiota (Hong *et al*., 2011). *Roseburia intestinalis* and *E. rectale*, butyrate producers belonging to *Clostridium* cluster XIVa, also preferentially colonize mucins in an *in vitro* gut model (Van den Abbeele *et al*., 2013). In mice, the mucosal interfold regions are highly enriched in *Firmicutes*, mainly *Lachnospiraceae* and *Ruminococcaceae* (Nava *et al*., 2011; Li *et al*., 2015). Furthermore, the epithelium of the colon forms invaginations, called colonic crypts, where partial oxygen pressure as well as specific types and concentrations of host glycans can be found. These characteristics make the crypts a reservoir for mucin‐degrading bacteria like *Bacteroides fragilis* (Macfarlane and Gibson, 1991; Lee *et al*., 2013). It should be noted, however, that differences between lumen and mucosal communities tend to be relatively small, which is likely due to extensive mucus shedding and mixing in the lumen.

The ability to metabolize the glycans and peptide backbone of mucin glycoproteins is likely to be a key factor in determining which microorganisms physically associate with the mucus layer. Non‐mucolytic bacteria, however, are also found in the mucus layer (Li *et al*., 2015), and may use this niche either merely as a physical habitat or by scavenging partially‐degraded mucins cleaved by mucolytic organisms. Both commensal and pathogenic organisms that do not possess machinery for degradation of mucins can benefit from host‐derived nutrients by taking advantage of glycan subunits liberated by specialist degraders (Li *et al*., 2015). The glycans that decorate mucin are highly sulfated and desulfation of mucins may also support the growth of sulfate‐reducing bacteria (SRB) such as *Desulfovibrio* spp. (Willis *et al*., 1996; De Weirdt and Van de Wiele, 2015) (Fig. 3). Sialyation of mucin glycans is also observed along the large intestine, and liberation of sialic acid by *B. thetaiotaomicron*, which secretes a sialidase but lacks the capacity to metabolize sialic acid, supports the growth of pathogens such as *Clostridium difficile* and *S*. Typhimurium (Ng *et al*., 2013). Similarly, *E. coli* was found to replicate preferentially in the mucus during re‐growth after antibiotic treatment, possibly due to the utilization of sialic acid and other mucosal monosaccharides (Wadolkowski *et al*., 1988; Chang *et al*., 2004). Some mucin degraders, however, have developed strategies to overcome competition with non‐mucolytic species for cleaved products. *Ruminococcus gnavus*, for example, produces an intramolecular trans‐sialidase that acts on mucin and other glycoproteins, releasing 2,7‐anhydro‐Neu5Ac instead of sialic acid (Tailford *et al*., 2015), which it can utilize but many other species cannot.

Interestingly, genetically‐dictated changes in the host mucus carbohydrate landscape can impact the gut microbiota. The FUT2 gene encodes an α‐1,2‐fucosyltransferase responsible for the fucosylation of secreted mucin glycans and lack of a functional copy of this gene alters the composition of the gut microbiota (Rausch *et al*., 2011; Kashyap *et al*., 2013). The gut microbiota of mice lacking fucosylated host glycans have reduced alpha diversity and decreased levels of members of the order *Clostridiales* (Kashyap *et al*., 2013). In humans, an unclassified species belonging to the family *Lachnospiraceae* was also identified as indicator species in individuals lacking a functional FUT2 gene (Rausch *et al*., 2011). Remarkably, germ‐free mice do not maintain ileal fucosylation after weaning, but colonization with the microbiota from conventionally‐housed mice restores it (Umesaki *et al*., 1981). This suggests a feedback loop between members of the microbiota and the host, whereby some commensal species can induce host secretion of specific nutrients and benefit from these nutrients. This is also possibly a mechanism for host selection of certain species that may have been refined over long‐term co‐evolution (Schluter and Foster, 2012). Not all bacteria are capable of inducing fucosylation. Mono‐colonization of mice with segmented filamentous bacteria (SFB) or *Bacteroides thetaiotaomicron* induces fucosylation, while *Lactobacillus murinus* does not (Umesaki *et al*., 1995; Bry *et al*., 1996; Goto *et al*., 2014). Both SFB and *B. thetaiotaomicron* can live in the mucus layer, in close proximity with the host epithelium. Interestingly, while *B. thetaiotaomicron* is able to induce fucosylation, a mutant unable to use L‐fucose as a carbon source is much less effective in inducing fucosylation (Bry *et al*., 1996), suggesting that the host may be able to sense and respond to fucose levels in the lumen.

## Fine‐scale spatial structuring

Freter postulated that in addition to competition for nutrients, competition for adhesion sites may also play an important role in survival in the intestines (Freter *et al*., 1983a, 1983b). This idea was based on the observation that *E. coli* could persist if they were the first colonizers of germ‐free mice and then a complex conventional microbiota was introduced, but *E. coli* could not establish in mice that had already been colonized by a conventional microbiota. While Freter's interpretation of this phenomenon was that there was competition for free adhesion sites on the intestinal wall (Freter *et al*., 1983b), this could also be interpreted as a priority effect in which there is an advantage in colonizing first and establishing a large population size before the introduction of other functionally‐similar species (Fukami, 2015). For example, colonization of antibiotic‐treated hamsters with non‐toxigenic *Clostridium difficile* protects against a subsequent challenge with epidemic *C. difficile*, most likely due to the occupation of the vacant nutrient niche by the non‐epidemic strain (Nagaro *et al*., 2013). More recently, however, Leatham and co‐authors reported that the mouse intestine selects for non‐motile *E. coli* that have improved growth on mucosal sugars, but that the non‐motile population does not completely outcompete motile *E. coli in vivo* (Leatham *et al*., 2005; Leatham‐Jensen *et al*., 2012). The remaining motile population had mutations in the gene coding for EnvZ, a kinase which together with OmpR forms a two‐component signal‐transduction system that regulates outer membrane protein profiles (Leatham‐Jensen *et al*., 2012; Adediran *et al*., 2014). Remarkably, the *envZ* mutant prevents expansion of non‐motile *E. coli* in di‐associated mice, suggesting that the mutation confers higher affinity for certain adhesion sites and that *E. coli* can reside in the mucus layer in mixed biofilms scavenging simple sugars released locally by other organisms (Leatham‐Jensen *et al*., 2012; Adediran *et al*., 2014). These studies led to the development of the ‘Restaurant hypothesis’, which states that organisms with the same nutritional preferences can co‐exist if they are part of spatially‐distinct biofilms where they obtain nutrients locally (Figs 1C, 2C and 3) (Leatham‐Jensen *et al*., 2012; Adediran *et al*., 2014; Conway and Cohen, 2015).

The Restaurant hypothesis suggests that fine‐scale spatial structuring would be important to overall ecosystem diversity (Fig. 1C). Analyses of human biopsies have found distinct mucosal‐associated microbiota not only along the length of the large intestine (Zhang *et al*., 2014), but also in biopsies collected only one centimetre apart (Hong *et al*., 2011), supporting the idea of heterogeneity in mucus‐associated communities on a small spatial scale. Fine‐scale spatial structuring also likely exists in luminal communities, as a high level of spatial heterogeneity and discrete patches have been observed in selected bacteria in feces (Swidsinski *et al*., 2008) (Fig. 3). Patchiness in feces may be due to aggregates of interacting microorganisms, micro‐environments originating from detached mucus, or heterogeneity of nutrient availability due to dietary fibres. The insoluble and liquid fractions of human feces have distinct microbiota and there is a specialized community associated with food particles (Walker *et al*., 2008). In homogenized diets, such as powder diets used for laboratory mice, there is a reduction of diversity in the microbiota compared to the same diet in pellet form (Clavel *et al*., 2014). Thus, dietary fibre seems to promote microbial diversity both by providing nutrient niches as well as creating spatial structure, indicating that the Restaurant hypothesis may also be applicable to luminal communities.

## No microbe is an island: interactions and dependencies in nutrient niches

Functional redundancy, or the co‐existence of functionally‐similar organisms, is often considered to be an important feature of the gut ecosystem that contributes to robustness and resilience (Moya and Ferrer, 2016). However, some key metabolic activities may be restricted to one or few species, called ‘keystone’ species or taxa. A keystone species has a large impact on the rest of the community (Figs 1E and 2E), and has sometimes also been defined as having a disproportionately low abundance relative to its impact on the ecosystem (Paine, 1966; Paine, 1969; Mills *et al*., 1993), though the low abundance criterion is not always applied. For example, degradation of dietary compounds such as starch by amylases of *R. bromii* leads to increased starch utilization by a number of other gut species (Ze *et al*., 2012). Because many community members depend on this primary starch degrader to provide them with soluble growth substrates, *R. bromii* is thought to be a keystone species. Co‐culture of *Akkermansia muciniphila*, a mucus degrader, enhances growth of *Bacteroides vulgatus* in mucin as the sole carbon source, thus functioning as a keystone species in the utilization of mucosal compounds (Png *et al*., 2010). Hydrogenotrophic organisms, which are largely dependent on the activity of fermenters for hydrogen and other compounds such as sulfate and sulfite cleaved from dietary‐ and host‐derived compounds, may also be keystones due to their ability to modulate hydrogen levels and thereby affect the activity of fermentative organisms and the energy extraction efficiency of the entire community (Carbonero *et al*., 2012; Rey *et al*., 2013).

Members of the *Bacteroides* are able to use a wide range of polysaccharides and a number of closely‐related species can co‐exist in the gut by cross‐feeding, resulting in complete polysaccharide utilization. For instance, *Bacteroides ovatus* releases glycoside hydrolases to break down inulin, but can utilize inulin without extracellular degradation (Rakoff‐Nahoum *et al*., 2016). Extracellular inulin breakdown by *B. ovatus*, however, allows *B. vulgatus* to use cleaved inulin products which would have been unavailable to it (Rakoff‐Nahoum *et al*., 2014; Rakoff‐Nahoum *et al*., 2016). *B. vulgatus*, in turn, increases *B. ovatus* fitness, presumably through detoxification of inhibitory substances or production of growth‐promoting factors. These secreted glycoside hydrolases can be viewed as public goods, yielding polysaccharide breakdown products that allow the growth of other organisms otherwise unable to grow on it. Production of public goods results in a complex polysaccharide utilization network that contributes to the creation of organized ecological units within the gut microbiota (Rakoff‐Nahoum *et al*., 2014; Rakoff‐Nahoum *et al*., 2016). The presence of insoluble substrates in the gut may serve as a scaffold to spatially organize public goods‐based interactions not only between the *Bacteroidales*, but also *Firmicutes* and other less abundant members of this ecosystem (Walker *et al*., 2008). Theoretical considerations suggest that dependencies and cooperation would be intrinsically unstable (Oliveira *et al*., 2014). This instability may be partially ameliorated by dependencies that can be fulfilled by many different organisms, such as the relationship between fermenters and hydrogenotrophs. Additionally, cooperation may be non‐obligate and the metabolic versatility of cooperators may allow them to switch their realized niche in the absence of their partner, thereby facilitating conditional cooperation (Figs 1E and 2E).

## Outlook: characterizing realized nutrient niches

Assembly of the gut microbiota is largely deterministic and driven by the nutrient landscape created by diet and host secretions. The realized metabolic niche of members of the microbiota is shaped both by nutrient availability as well as the presence of other competing or cooperating species and resulting niche partitioning (Figs 1 and 2). Freter's classic nutrient niche theory, which states that the abundance of each species is determined by a single limiting nutrient, is a conceptually useful model of assembly that has been supported by experimental work in gnotobiotic animal experiments. However, the validity of this model has been challenged by observations of mixed‐substrate utilization and metabolic flexibility as well as heterogeneity in nutrient levels in time and space. These processes weaken competitive exclusion and allow for increased diversity, which is likely critical for ecosystem robustness.

A deeper understanding of the *in situ* metabolic niche of individual members of the microbiota is needed in order to better comprehend the relative importance of the above‐mentioned processes. As metabolic strategies that define the realized niche are highly dependent on ecological context, this is best explored by direct analysis of the complex microbiota. To dissect these niches, specialized tools that allow for study of the activity of complex microbial communities should be applied. These include metatranscriptomics (Franzosa *et al*., 2014; Franzosa *et al*., 2015; Plichta *et al*., 2016), metaproteomics and metabonomics (Ferrer *et al*., 2013; Theriot *et al*., 2014; Franzosa *et al*., 2015), stable isotope probing (Tannock *et al*., 2014; Young *et al*., 2015), as well as single‐cell tools such as FISH and single‐cell stable isotope probing (Berry *et al*., 2013; Stecher *et al*., 2013; Berry *et al*., 2015) which can uniquely be used to study fine‐scale spatial heterogeneity in composition and activity. An improved understanding of nutrient niches and assembly of the gut microbiota will open the way to customized design of communities and novel therapeutic strategies to effectively modulate the microbiota to improve health.
